# A Systematic Review of Heavy Metals of Anthropogenic Origin in Environmental Media and Biota in the Context of Gold Mining in Ghana

**DOI:** 10.1155/2014/252148

**Published:** 2014-11-09

**Authors:** Frederick Ato Armah, Reginald Quansah, Isaac Luginaah

**Affiliations:** ^1^Environmental Health and Hazards Laboratory, Department of Geography, Western University, 1151 Richmond Street, ON, Canada N6A 5C2; ^2^Department of Environmental Science, School of Biological Sciences, University of Cape Coast, Cape Coast, Ghana; ^3^Biological, Environmental & Occupational Health Sciences, School of Public Health, University of Ghana, Legon, Accra, Ghana; ^4^Department of Geography, Western University, 1151 Richmond Street, ON, Canada N6A 5C2

## Abstract

Heavy metal accumulation in the food chain is an issue of global concern because it eventually leads to toxic effects on humans through the water we drink, contaminated soils, crops, and animals. Reports of toxicant levels in environmental media (air, water, and soil) and biota in Ghana were sought in SCOPUS, PubMed, MEDLINE, and EMBASE. Of 1004 bibliographic records identified, 54 studies were included in evidence synthesis. A disproportionately large number of papers (about 80%) focused exclusively on environmental media. Papers focusing on biomonitoring and human health were relatively few. Studies reported a high degree of spatial variability for the concentrations of 8 metals in groundwater. Generally, heavy metal concentrations in soil reported by the studies reviewed were higher than metal concentrations in riverine sediments. Urine and hair were the most common biological markers of heavy metal exposure used by the studies reviewed unlike nails, which were sparingly used. By and large, published results on the levels of heavy metals in goldmine and non-mine workers yielded contradictory results. Mostly, concentrations of heavy metals reported by the studies reviewed for nails were higher than for hair. A high degree of variability in the heavy metal concentrations in human subjects in the studies reviewed is likely due to heterogeneity in physiological states, excretion profiles, and body burdens of individuals. These, in turn, may be a product of genetic polymorphisms influencing detoxification efficiency.

## 1. Introduction

Over the past three decades, the term “heavy metals” has been widely used in the scientific literature on ecotoxicology. It is frequently considered as an assemblage name for metals and semimetals (metalloids) that have been linked with contamination and potential toxicity or ecotoxicity [1]. The term “heavy metals” has, however, been used inconsistently in the scientific literature. This has culminated in considerable misperception of the significance of the term. There is also a propensity to suppose that all the so-called “heavy metals” have highly toxic or ecotoxic properties [1]. According to Duffus [1], the scientifically sound designations of elements generally considered as heavy metals are as follows: As, Cd, Hg, Pb, and Sb (Chalcophile); Fe, Co, Cu, Ni, and Zn (lithophile/chalcophile); and Mn and Cr (lithophile). Despite some recognition that the term “heavy metals” is a misnomer, we resort to its use in this paper for two fundamental reasons. First, although the term “heavy metals” has been queried over many years, for example, by Phipps [2], and by Loon and Duffy [3], efforts to replace it by chemically sound terminology have so far failed. Secondly, all the papers systematically reviewed in this study specifically used the term “heavy metals.”

Heavy metals in water, sediments, air, and other environmental media are of great environmental concern because of their potential long-term effects on human health particularly in developing countries where remedial techniques are nascent [4–8]. The origin of such metals in the natural environment is either geogenic or anthropogenic releases [9, 10]. In general, the anthropogenic releases constitute a constant source of pollution, whereas surface runoff is a seasonal phenomenon which is influenced by climate within the environmental system. The concentrations of heavy metal (loid)s in soils and other environmental media can vary widely, even in uncontaminated environments. Noticeable dissimilarities in the geochemical composition of the rocks which constitute the parent materials of soils and disparities in the strength of soil-forming processes can lead to extensive ranges of total and available concentrations of most elements in soils, even in those unaffected by contamination [4]. Nevertheless, contamination from many sources can often give rise to some very high concentrations of heavy metal (loid)s [4] which can cause toxicity in soil organisms and susceptible plants, but this depends on the factors affecting the bioavailability of the elements [4]. Many investigations have been conducted on anthropogenic contaminants of ecosystems across the globe [11, 12].

In Ghana, which exemplifies a country where extensive research on heavy metals has hitherto been carried out because of its extensive mining industry, one of the main anthropogenic sources of concern is gold mining, both surface and underground. Gold mining is widespread and according to Naylor [13], it contributes about 44% of Ghana's export earnings. The large-scale extraction of gold occurs predominantly in the Western and Ashanti regions for example, Bibiani and Obuasi, and is accompanied by arsenic, mercury, and sulphur contamination to surface and groundwater bodies, soil and even air pollution causing acid rain and degradation to the surrounding environment and impacts on human health [14, 15]. With the liberalization of the gold mining sector in the mid-1980s, gold mining-dependent livelihoods have soared, employing extraction methods that invariably release mercury into surrounding water sources [5, 16, 17]. In the past, gold mining was restricted to the south; lately however, exploration is increasing in the north, especiallyin the Upper East Region. Iron and manganese have also been found in elevated concentrations in water in Ghana [9]. This has culminated in the closure of hundreds of wells in favour of surface waters likely contaminated with harmful microorganisms [18]. Up till now, mining related studies in Ghana conducted on environmental samples (water, air, soil, sediment, etc.) and biota (fish, urine, blood, nails, etc.) include Hg [19, 20], As [14, 15], Fe and Mn [9, 21], Pb [7, 18], and Cd and Cu [5, 16, 17].

Despite the large body of literature that has been devoted to heavy metal pollution in Ghana, the results are mixed and are disparate making it quite difficult to elicit a coherent account on the scope and levels of heavy metal pollution in the environment and in biota, especially in humans, across Ghana. Consequently, this study aims to cumulate scientific evidence on heavy metal pollution in the environment and in biota in Ghana through synthesis of existing data. This systematic review was conducted for a variety of reasons, but it was not limited to the synthesis of evidence on the magnitude of heavy metal contamination or to supporting evidence-based policy or practice. This review provides useful information for designing future research on heavy metal pollution in Ghana and other jurisdictions. In particular, it will help to place future studies in context by describing what we knew before and what we hoped to learn from any future study on heavy metal pollution in Ghana and in other contexts.

## 2. Materials and Methods 

### 2.1. Search Strategy and Study Selection

The systematic search and review processes were conducted in accordance with the Preferred Reporting Items for Systematic Reviews and Meta-Analyses (PRISMA) Statement criteria as proposed by Liberati et al. [22]. We searched SCOPUS, PubMed, MEDLINE (http://www.ncbi.nlm.nih.gov/pubmed/), and EMBASE (http://www.embase.com/home) databases using the following search terms: “Ghana” successively combined with “heavy metals,” “pollutant,” “toxic element,” “metalloid,” “arsenic,” “cadmium,” “mercury,” “lead,” “cobalt,” “zinc,” “manganese,” “iron,” “nickel,” and “chromium.” The search was limited to papers published till January 2013 and yielded 1004 bibliographic records. The bibliographic records were complemented with attempts of search for other research by key authors and search of citations and reference lists of key reports and related articles. After importing bibliographic records duplicates were deleted and reports were scrutinized using Zotero 2.03. All studies presenting quantitative data on levels of arsenic, cadmium, lead, mercury, chromium, cobalt, nickel, manganese, iron, tin, and zinc in environmental media (soil, water, sediment, and air) and or biologic specimen (hair, urine, blood, nails, and food) were included, regardless of experimental design, or methods of collection of samples, or chemical analysis.

### 2.2. Data Extraction

Using a standard, purpose-designed form, we extracted the following data from each paper: (a) study design, date and place, sampling method and size, inclusion and exclusion criteria, and request for informed consent; (b) protocol for collection, storage, processing, and analysis of biologic specimens; and (c) results, including metal levels and related factors.

## 3. Results 

### 3.1. Description of the Studies

**Figure 1:**
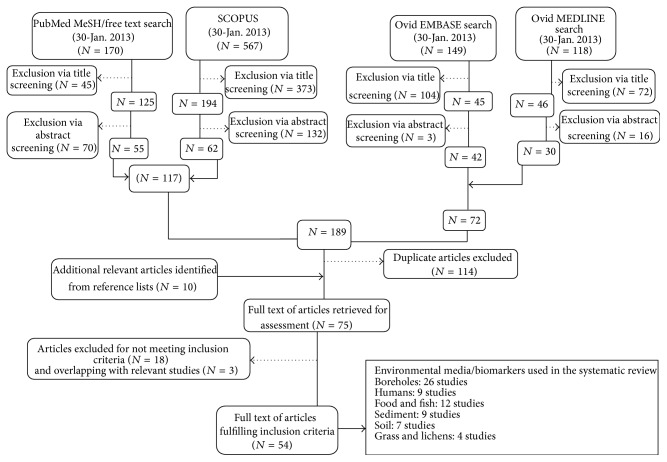
Schematic diagram of search strategy.

Of 1004 bibliographic records of relevance to the gold mining context in Ghana identified, 594 did not meet inclusion criteria at first screening, 83 full-text articles were sought for full-text screening, and 65 were obtained and screened. Fifty-four studies were included in evidence synthesis. Searching, screening, and study inclusion are summarized in the flow diagram, Figure 1.

The main characteristics of each study are described in Tables 3, 4(a)–4(c), and 5(a)–5(e). Whereas older studies (from mid 1970s through early 1990s) on heavy metals in environmental media and biologic specimens in Ghana were mainly undertaken along the Ashanti gold belt in south western Ghana (to a large extent in Obuasi and to some extent in Tarkwa), studies targeting environmental media in the northern parts of Ghana began to be published only in the early 2000s and accounted for an ample fraction (20%) of published works from 2000 and later. Of the reviewed articles, the earliest study on heavy metals in environmental media and biologic specimens in Ghana was undertaken by Simeonov et al. [23]. Thereafter, there was an almost twenty-year lull in research on heavy metals before the work of Amonoo-Neizer and Amekor [14].

### 3.2. Research Design and Objectives

All the papers reviewed were cross-sectional studies with three distinct types of objective, often combined in the same study, that is, assessment of levels of heavy metals in the media, spatial variability of the metals, and compliance with environmental and regulatory standards. None of the studies monitored heavy metal concentrations in environmental media or biologic specimens across time (longitudinally). Broadly, studies adopted either an environmental monitoring or a bio-monitoring perspective. A disproportionately large number of papers (about 80%) focused exclusively on environmental media. Out of the 54 articles reviewed, twelve papers devoted attention to heavy metals in either cooked (fish) or uncooked food (vegetables, fruits) or other plants (lichens).

Papers focusing on biomonitoring and human health were few and far between. In total, 10 articles focused on heavy metals in humans. As shown in Tables 1(a) and 1(b), six papers analysed heavy metals in human hair. Eight manuscripts measured heavy metals in human urine and only two articles focused on heavy metals in human blood. Also, two measured heavy metals in human nails. Of the studies reporting on human subjects, only one reported on 1 biomarker of exposure (hair), whereas another study reported on three biomarkers (hair, blood, and urine). The rest reported on at most two biomarkers (see Tables 1(a) and 1(b)). Regarding ethical considerations, 6 papers on human subjects specifically mentioned informed consent as a prerequisite for study participation and 2 papers, mostly recent, stated the approval of an ethics committee.

### 3.3. Analytical Methods: Collection, Processing, and Analysis of Biologic Specimens

Period of collection of environmental samples and biologic specimens, which was not always specified, varied extensively as shown in Table 3. For instance, more than 20% of articles reviewed did not report period of data collection (11 papers). Similarly, pretreatment of environmental samples, which was not always specified, varied widely. In general, the environmental and biologic samples (water, urine, blood, etc.) were frozen and stored before laboratory analyses. A variety of spectrometry was used to determine levels of heavy metals. These include UV-visible spectrophotometry (2 papers), cold vapour atomic absorption spectrophotometry (5 papers), instrumental neutron activation analysis (10 papers), inductively coupled plasma mass spectrometry (ICP-MS) (4 papers), and inductively coupled plasma-optical emission spectroscopy (ICP-OES) (1 paper). Other techniques included flame atomic absorption spectrophotometry (12 papers), high performance liquid chromatography (HPLC) (1 paper), inductively coupled plasma atomic emission spectrometer (ICP-AES) (1 paper), and atomic absorption spectrophotometry (6 papers). Five studies did not report the analytical method used in the determination of heavy metals in environmental media or biologic samples.

Information on laboratory quality controls differed between the earliest and the more recent papers. Before the year 2000, none of the studies specified having internal quality controls or external controls. In contrast, all studies published after 2005 (7 papers) reported the use of standardized quality control procedures that generally comprised evaluation of accuracy and precision by analysis of certified reference material; however, no studies included additional inter-laboratory comparisons. Only about one-third of the studies specified the limit of detection (LOD), merely stating the value or including the procedure used to treat values below it.

### 3.4. Statistical Analyses and Reporting of Results

Most of the studies (>90%) provided measures of central tendency, that is, arithmetic means, usually accompanied by standard deviations (SDs). Geometric mean was not reported in any of the papers. Other widely used indicators of dispersion and central tendencies were range (more than 40 studies) and median (15 studies). Without exception, authors neither include confidence intervals nor mention the evaluation of outliers or the use of robust measures of central trend. Studies assessing associations with risk factors generally provided either Pearson's product moment or Spearman's correlation coefficients drawn from univariate analyses or differences in means derived from stratified analyses; few papers (2 papers) carried out multivariate analyses.

### 3.5. Levels of Heavy Metals in Underground Water and Water from Boreholes

Studies reported a high degree of spatial variability for the concentrations of 8 metals (As, Hg, Cd, Cr, Pb, Co, Sr, and Mn) in boreholes as shown in Table 2. The lowest concentration of As (<1 *μ*gL^−1^) was reported by Akabzaa et al. [24] for the Anglogold Ashanti area in Obuasi. The highest concentration of As (12200 *μ*gL^−1^) was reported by Boadu et al. [25] for the Konongo Old mining shaft. The lowest concentration of Hg (<0.05 *μ*gL^−1^) was reported by Asante et al. [15] for the Tarkwa gold mining area, whereas the highest Hg concentration of 6251 *μ*gL^−1^ was reported by Essumang et al. [16] for the Wassa West District. The lowest concentration of Cd (<0.06 *μ*gL^−1^) was reported by Asante et al. [15] for the Tarkwa gold mining area and the highest Cd concentration of 7.6 *μ*gL^−1^ was reported by Tay and Momade [26] for the northern part of the Ashanti gold belt. The lowest concentration of Cr (0.03 *μ*gL^−1^) was reported by Asante et al. [15] for the Tarkwa gold mining area, whereas the highest Cr concentration of 45 *μ*gL^−1^ was reported by Essumang et al. [16] for the Dumasi community in the Wassa West District. Three studies [15, 24, 27], severally reported the lowest Pb concentration of 0.01 *μ*gL^−1^. However, Akabzaa et al. [24] reported the highest Pb concentration (96 *μ*gL^−1^) within the vicinity of the Obuasi goldmine. The lowest concentration of Co (0.02 *μ*gL^−1^) was reported by Asante et al. [15] for the Tarkwa gold mining area, whereas the highest Co concentration of 50 *μ*gL^−1^ was reported by Essumang et al. [16] for the Dumasi community in the Wassa West District.

### 3.6. Levels of Heavy Metals in Riverine Sediments and Soil

There was a high degree of variability in metal concentrations in riverine sediments and soil reported by the studies reviewed (Table 4). The lowest concentration of As in sediment was reported by Akabzaa et al. [24] for the Anglogold Ashanti area in Obuasi and the highest As concentration (10,200 mgkg^−1^) was reported by Serfor-Armah et al. [28] for Prestea in the Western region of Ghana. The lowest concentration of Hg (0.01 mgkg^−1^) was reported by Boamponsem et al. [29] for Teberebie spring 3 in the Tarkwa gold mining area. Also, Boamponsem et al. [29] reported the highest concentration of Hg (200 mgkg^−1^) for bottom sediments of the Offin River basin. The highest concentration of Pb (115 mgkg^−1^) was reported by Akabzaa et al. [24] for communities immediately downstream of the Anglogold Ashanti mine in Obuasi.

Generally, heavy metal concentrations in soil reported by the studies reviewed were higher than metal concentrations in riverine sediments. Studies reviewed did not report on Mn, Ni, or Pb concentrations in soil. The lowest As concentration (0.7 mgkg^−1^) in soil was reported by Hayford et al. [30], whereas the highest As concentration (2875 mgkg^−1^) in soil was reported by Amasa [31]. The lowest concentration of Hg (mgkg^−1^) in soil was reported by Oppong et al. [32] for the Pra River basin at Daboase, Western Ghana. The highest concentration of Hg (2146 mgkg^−1^) was reported by Donkor et al. [33] for the Offin River basin.

### 3.7. Levels of Heavy Metals in Fruits, Vegetables, and Edible Plants

Generally, studies reviewed focused mainly on the chemical content of certain elements in the biologic specimen. None focused on the influence of heavy metals on the morphological or cellular structure or metabolic-biochemical processes in the biologic specimen. Some of the studies categorised the food items analysed into cooked and uncooked (e.g., [14]), whereas others did not distinguish between the cooked and uncooked forms [24]. Only one study [34] reported on heavy metals using lichens as biologic specimen in the Obuasi gold mining area. Similarly, one study [14] used star grass as biologic specimen. However, two studies [19, 31] used ferns and two other studies [14, 19] used elephant grass as biologic specimen. Several fruits and vegetables were used in the studies reviewed as biologic specimen. The vegetables include pepper, beans, cocoyam, cassava, and plantain. Fruits include oil palm, cocoa, sugar cane, pear, orange, and banana.

Amasa [31] reported very high concentration of As in ferns (up to 4700 mgkg^−1^) and oil palm (2900 mgkg^−1^). The lowest As concentrations were reported for plantain (2.29 mgkg^−1^) and cassava (2.65 mgkg^−1^) in Obuasi and its environs (Tables 5(a)–5(e)). Generally, the magnitude of As concentrations in soil and biologic specimen in decreasing order as reported by the studies reviewed was as follows: palm tree > fern > soil > sugar cane > banana > orange > cocoyam > cassava > plantain. Essumang et al. [16] reported As concentrations as high as 383 in water cocoyam grown in soils of the Tarkwa gold mining area. The magnitude of Hg concentrations in soil and biologic specimen in decreasing order as reported by the studies reviewed was as follows: fern > soil > elephant grass > plantain > cassava.

### 3.8. Levels of Heavy Metals in Urine and Blood

Studies reviewed broadly reported heavy metals concentrations in urine for gold mine workers, non-gold mine workers, and workers exposed to fumes from e-waste recycling. Urine was the most common biological marker of heavy metal exposure used by the studies reviewed. Generally, reports on the levels of heavy metals in gold mining workers and their non-gold mining counterparts did not reveal any discernible pattern. For instance, from Asante et al. [15] it can be deduced that the non-mine worker/mine worker ratios for As, Hg, Mn, Cd, Zn, Cr, Cu, and Pb were 1.2, 10.9, 1.3, 7.7, 0.3, 3.7, 1.4, and 1.2, respectively. This suggests that except Zn non-mine workers generally had lower concentrations of heavy metals in their urine compared to their counterparts who were mine workers. This result is counter-intuitive. Heavy metals in urine samples of small scale artisanal gold miners in the Upper East region did not differ significantly from heavy metals in urine samples of e-waste recyclers in Accra. Only one study [35] reported on heavy metals in blood samples mostly in some residents in the Western region of Ghana. Mercury levels in such residents varied spatially. For instance, Adimado and Baah [35] reported Hg concentrations of 218 *μ*gL^−1^ and 57 *μ*gL^−1^ for residents in Bibiani Anhwiaso Bekwai and Tanoso, in south western Ghana.

### 3.9. Levels of Heavy Metals in Hair and Nails

Hair was the second most common biological marker of heavy metal exposure used by the studies reviewed unlike nails, which were sparingly used. Generally, concentrations of heavy metals reported by the studies reviewed for nails were higher than for hair. One study [35] reported higher concentrations of heavy metals in nails than in hair.

## 4. Discussion 

In this review, we provide a systematic categorisation of the results of studies published from 1975 to January 2013 on As, Hg, Cd, Zn, Sb, Cr, Fe, Co, Cu, Ni, Zn, Mn, and Pb levels in water, soil, sediment, fruits, and vegetables as well as human hair, urine, blood, and nails in Ghana. We found that the use of vegetables, fruits, fish, hair, nails, and blood as biomarkers of exposure to heavy metals is not appropriately developed because of the heterogeneity among the studies. This heterogeneity applies not only to the populations selected and the analytical techniques (as it impinges on accuracy and precision) but also to the processing of specimens and presentation of results. The procedure for collection, pretreatment, storage, and preparation of the heavy metals prior to analysis varied extensively among studies, although available data suggest that such processes may influence the magnitude and comparability of the trace metal concentrations [36, 37].

It would appear that large variability in measured concentrations, apart from collection methods as mentioned above, was likely the result of differences in spatial characteristics of the sampling locations. Similarly, for the published studies that focused on human subjects, the variability in measured concentrations may emanate from varying excretion profiles amongst widely varying individuals with ranges of body burdens, genetic polymorphisms affecting detoxification efficiency, and physiological states [38]. These variations were very much greater than would be expected due to limitations of analytical methods. Although analytical methods have improved over the years, analysis of these metals was routine at the time of the studies.

Apart from geogenic sources, the contamination chain of heavy metals emanating from anthropogenic sources almost always follows a cyclic order: industry, atmosphere, soil, water, foods, and humans [39]. Regarding exposure to contaminants in environmental media, surface and groundwater were the most studied. Published results clearly indicate widespread contamination of ground and surface water especially in gold mining environments. Groundwater quality in natural systems is a result of many environmental factors. Climate, geology, biochemistry, composition of atmospheric precipitation, and the nature of the hydrology are among the more important factors [39].

Arsenic concentrations in aquifers in different geologic settings vary considerably over short distances. In the mining communities the geologic settings are Tarkwaian or Birimian systems [40]. Arsenic in groundwater occurs in two species (chemical forms), As (III) and As (V), which denote the As oxidation state. Arsenic (V) consists of arsenic acid (H_3_AsO_4_) and its conjugate bases (H_2_AsO_4_^−^, HAsO_4_^2−^, and AsO_4_^3−^). In the pH range of most natural waters, the predominant As (V) species are the anions H_2_AsO_4_^−^ and HAsO_4_^2−^ [39]. In the same pH range, As (III) consists of mostly uncharged arsenious acid (H_3_AsO_3_) with a minor amount (<10%) of the anion H_2_AsO_3_^−^ which sorbs As (III) [39, 41]. The predominant As species in groundwater in the mining communities (i.e., Bogoso, Tarkwa, Teberebie, Damang) is As (III), which is obtained from the oxidation of FeAsS (arsenopyrite) ore abundant in these communities [40]. Most of ingested arsenic is rapidly excreted via the kidney within a few days. However, high levels of arsenic are retained for longer periods of time in the bone, skin, hair, and nails of exposed humans [36].

Iron concentration in groundwater varied extensively with location. The inconsistent value of Fe obtained in this study is not unexpected owing to the high occurrence of the metal in nature. Fe constitutes a high weight percentage in sulphide ores and is therefore likely to be very high, at areas of active metallurgic activity such as Tarkwa, Damang, and Bogoso. According to Kelly et al. [41], in wells for which the total organic carbon (TOC) concentration is less than ~2 milligrams per liter (mg/L), As is usually undetectable (<1 *μ*g/L). For wells with higher TOC values, high As concentrations are more likely. In wells with detectable sulfate, As is almost always undetectable, while wells with undetectable sulfate may have high As concentrations. A likely explanation is that As is associated with iron oxide coatings on sand grains in the aquifer [39]. In areas where organic carbon is abundant, the iron oxide gets reduced and the As is released to the groundwater. In areas with abundant sulfate, sulfate reduction forms ferrous sulfide (FeS), and the predominant As (V) species are the anions H_2_AsO_4_^−^ and HAsO_4_^2−^ [39, 42].

Levels of Cd in groundwater demonstrated that weathering of calcareous rocks was sufficient to provide the amounts of Cd found in the water profiles. In addition, the input of Cd by weathering was larger than the input by anthropic or geogenic atmospheric depositions which are the only other potential sources of Cd in mining areas. Several published studies reviewed reported Cd in human hair and nails. Cadmium accumulates in the human body adversely affecting a number of organs: liver, kidney, lung, bones, placenta, brain, and the central nervous system [43]. According to Apostoli and Catalani [44], other cadmium-related health effects that have been observed include reproductive, and development toxicity, hepatic, haematological and immunological effects.

According to Morais et al. [36], tobacco smoke is one of the largest single sources of cadmium exposure in humans. Eugenio Figueroa [45] argues that given the fact that the absorption of cadmium from the lungs is much greater than from the gastrointestinal tract, smoking contributes significantly to the total body burden. On the whole, for nonsmokers and non-occupationally exposed workers, food products comprise most of the human exposure burden to cadmium [36]. Some of the published studies reviewed reported Cd in a variety of food items including fruits [24], cocoyam [16], and shell and fin fish species [46, 47]. In food, only inorganic cadmium salts are present. Organic cadmium compounds are very unstable. Unlike lead and mercury ions, cadmium ions are readily absorbed by plants. They are evenly distributed over the plant. It is widely known that cadmium is taken up through the roots of plants to edible leaves, fruits, and seeds. In fact, during the growth of grains such as wheat and rice, cadmium taken from the soil is concentrated in the core of the kernel [45]. Cadmium also accumulates in animal milk and fatty tissues [45]. Therefore, individuals are exposed to cadmium when eating plant- and animal-based foods. As argued by Castro-González and Méndez-Armenta [43] seafood, such as molluscs and crustaceans, can be also a source of cadmium.

The principal controls on manganese concentration in groundwater are pH (acidity) and redox (oxidation-reduction) condition. Manganese is mobilised under acidic conditions. Hence concentrations can be relatively high in acidic waters such as some industrial waters and those issuing from mines rich in weathered sulphide minerals. This resonates with the situation in the mining areas under study. In pH-neutral conditions, the mobility of manganese is determined by ambient redox conditions. Under anaerobic conditions, manganese is reduced to the more soluble form, Mn(II), which is released from minerals. As a result, much higher manganese concentrations can be found in anaerobic ground waters.

The highest maximum dissolved Pb concentrations were found in water samples from surficial aquifer systems, which is not surprising given the highly corrosive conditions (typically low pH, high DOC concentrations, and low dissolved oxygen levels). Lead most likely is transported in ground water by mobile particulate matter [48]. Erel et al. [49] estimate that up to 15 percent of the industrial Pb deposited from atmospheric deposition is incorporated in water that infiltrates through soils to ground water. Other studies have demonstrated a downward migration of Pb through soils and into aquifers, which depends on the mobility of organic matter and sesquioxides because of the high stability of Pb-organic matter and Pb-sesquioxide complexes [50]. Any dissolved Pb that is present (e.g., low pH waters) in ground water would tend to form complexes with several anionic ligands and the migration of dissolved Pb in ground water would be dependent on its form (the predominant Pb species) in solution. For example, free divalent Pb ion (Pb^2+^), which is the predominant species of Pb in low-ionic strength waters, tends to sorb on negative sites of clays and other minerals and aquifer materials [51]. Lead concentrations in ground water are related to differences in chemical conditions among aquifers and aquifer systems. In humans, lead ingestion may arise from eating lead contaminated vegetation or animal foods. Another source of ingestion is through the use of lead-containing vessels or lead-based pottery glazes [36].

It is not always that existence of these metals in humans should be considered as toxic. In higher animals and humans, the proven micronutrients include Cr, Cu, Fe, Mn, and Zn. There is even some evidence that Cd, Pb, and Sn may be essential at very low concentrations [4, 52, 53] although this evidence is disputed by Vieira et al. [54] and Morais et al. [36]. However, the micronutrients which have been conclusively proven to be essential in animal and/or human nutrition and whose concentrations in diets are critical are Co (ruminants only), Cr, Cu, Fe, Mn, and Zn [4].

Published results on the levels of heavy metals in mine and non-mine workers yielded contradictory results. Mine workers were anticipated to have higher levels of heavy metals than their non-mining counterparts, but published results did not support this expectation. In fact, two of the studies reviewed found evidence to the contrary.

## 5. Conclusions 

There is a plethora of environmental issues and concerns on which many scientists have focused their research in past years. In Ghana, tremendous efforts have been mobilized to evaluate the nature, presence, magnitude, fate, and toxicology of anthropogenic-induced heavy metals in diverse environments. The scope of this list is quite broad, encompassing environmental events locally, regionally, and nationally. Heavy metals affect aquatic and terrestrial ecosystems and biotic and abiotic environments and impacts on plants, humans, and wildlife, and virtually all environmental media (soil, water, and air). The staggering volume of scientific literature (during the last half century) on heavy metal contamination of environmental media and biota in mining and non-mining environments in Ghana demands remedy by which data can be synthesized. There is thus an urgent need to provide the coherency essential for nonduplicative and current progress in this field which is dynamic and complex. This systematic review attempts to address this need and provides a systematic categorisation of the results of studies published from 1975 to January 2013 on As, Hg, Cd, Zn, Sb, Cr, Fe, Co, Cu, Ni, Zn, Mn, and Pb levels in water, soil, sediment, fruits, and vegetables as well as human hair, urine, blood, and nails in Ghana.

## Figures and Tables

**(a):** 

Reference (sample type)	Sample type/region/community	As	Hg	Fe	Mn	Cd	Zn	Cr	Cu	Pb
Amasa 1975 [31]	Hair/Ashanti region/Obuasi mine workers (PTP)	336.33 (196–1940) mgkg^−1^	—	—	—	—	—	—	—	—
	Hair/Ashanti region/Obuasi mine workers (shaft)	25.59 (7.7–78.0) mgkg^−1^	—	—	—	—	—	—	—	—
	Hair/Ashanti region/Obuasi non-mine workers	38.7 (8.8–268) mgkg^−1^	—	—	—	—	—	—	—	—
Adimado and Baah 2002 [35]	Blood/western region/	—	102 (55.8)	—	—	—	—	—	—	—
	Urine/western region/	—	34.2 (36)	—	—	—	—	—	—	—
	Hair/western region/	—	1.61 (1.33)	—	—	—	—	—	—	—
	Nail/western region/	—	2.65 (2.0)	—	—	—	—	—	—	—
Essumang 2009 [55]	Hair/Western region/	0.0142–0.0515 *μ*gg^−1^	—	—	—	—	—	—	—	—
Paruchuri et al. 2010 [56]	Urine/Upper East region/	—	17.0 *μ*gL^−1^	—	—	—	—	—	—	—
Paruchuri et al. 2010 [56]	Hair/Upper East region/	—	1.1 *μ*gg^−1^	—	—	—	—	—	—	—
Basu et al. 2011 [57]	Urine/Upper East region/	114.52 *μ*gL^−1^	—	—	2.01 *μ*gL^−1^	0.45 *μ*gL^−1^	601.27 *μ*gL^−1^	26.58 *μ*gL^−1^	40.85 *μ*gL^−1^	1.34 *μ*gL^−1^
Abrefah et al. 2011 [58]	Urine/Western region/	14.75 *μ*gL^−1^	0.56 *μ*gL^−1^	—	—	—	—	—	—	—
Asante et al. 2012 [59]	Urine/Greater Accra region/	54.4 (45.6) *μ*gL^−1^	<0.5 *μ*gL^−1^	180 (150) *μ*gL^−1^	4.08 (2.39) *μ*gL^−1^	0.43 (0.17) *μ*gL^−1^	752 (520) *μ*gL^−1^	19 (7) *μ*gL^−1^	305 (117) *μ*gL^−1^	0.08 (0.01–0.08) *μ*gL^−1^
Adimado and Baah 2002 [35]	Blood/Western region/Anwiaso	—	102 (30.2–218) *μ*gL^−1^	—	—	—	—	—	—	—
Adimado and Baah 2002 [35]	Urine/Western region/Anwiaso	—	34.2 (1.0–183) *μ*gL^−1^	—	—	—	—	—	—	—
Adimado and Baah 2002 [35]	Hair/Western region/Anwiaso	—	1.61 (0.15–5.86) *μ*gg^−1^	—	—	—	—	—	—	—
Adimado and Baah 2002 [35]	Nail/Western region/Anwiaso	—	2.65 (0.57–10.0) *μ*gg^−1^	—	—	—	—	—	—	—
Asante et al. 2007 [15]	Urine (mine workers)/Western region/Tarkwa and its environ	70.6 (8–270) *μ*gL^−1^	0.35 (0.10–0.61) *μ*gL^−1^	—	1.56 (1.0–2.4) *μ*gL^−1^	0.03 (0.03–0.11) *μ*gL^−1^	1.71 (0.10–0.42) *μ*gL^−1^	8.15 (2.6–25.1) *μ*gL^−1^	561.07 (64.1–1230) *μ*gL^−1^	0.028 (0.01–0.08) *μ*gL^−1^
Asante et al. 2007 [15]	Urine (non-mine workers)/Western region/Tarkwa and its environ	83.85 (4.7–123) *μ*gL^−1^	3.84 (0.6–86.0) *μ*gL^−1^	—	1.98 (0.2–4.1) *μ*gL^−1^	0.23 (0.01–0.27) *μ*gL^−1^	0.54 (0.10–5.65) *μ*gL^−1^	29.78 (3.7–103) *μ*gL^−1^	792.18 (159–1600) *μ*gL^−1^	0.034 (0.01–0.26) *μ*gL^−1^
Asante et al. 2012 [59]	Urine (e-waste recycling workers)/Greater Accra region/Agbogbloshie	0.34 (0.10–0.76) *μ*gL^−1^	—	13 (5.3–21) *μ*gL^−1^	5.19 (1.18–10.0) *μ*gL^−1^	0.07 (<0.01–0.19) *μ*gL^−1^	94.2 (7.85–711) *μ*gL^−1^	0.38 (0.04–1.5) *μ*gL^−1^	20.8 (5.89–52.0) *μ*gL^−1^	0.40 (0.09–1.23) *μ*gL^−1^
Adimado and Baah 2002 [35]	Blood/Western region/Tanoso	—	16.5 (2.1–57.2) *μ*gL^−1^	—	—	—	—	—	—	—
Adimado and Baah 2002 [35]	Urine/Western region/Tanoso	—	6.4 (2.0–14.3) *μ*gL^−1^	—	—	—	—	—	—	—
Adimado and Baah 2002 [35]	Hair/Western region/Tanoso	—	4.27 (0.06–28.3) *μ*gg^−1^	—	—	—	—	—	—	—
Adimado and Baah 2002 [35]	Nail/Western region/Tanoso	—	3.45 (0.13–22.6) *μ*gg^−1^	—	—	—	—	—	—	—
Kwaansa-Ansah et al. 2010 [60]	Hair (Farmers)/Central region/Dunkwa-On-Offin	—	2.35 (0.63–7.19) *μ*gg^−1^	—	—	—	—	—	—	—
	Urine (Farmers)/Central region/Dunkwa-On-Offin	—	0.69 (0.075–2.31) *μ*gL^−1^	—	—	—	—	—	—	—
Kwaansa-Ansah et al. 2010 [60]	Hair (miners)/Central region/Dunkwa-On-Offin	—	2.14 (0.57–6.07) *μ*gg^−1^	—	—	—	—	—	—	—
	Urine (miners)/Central region/Dunkwa-On-Offin	—	1.23 (0.32–3.62) *μ*gL^−1^	—	—	—	—	—	—	—

**(b):** 

Reference (sample type)	Sample type/region/community	Ni	Co	Cr	Se	Sb	Sr	Rb
Basu et al. 2011 [57]	Urine/Upper East region/	6.51 *μ*gL^−1^	2.32 *μ*gL^−1^	26.58 *μ*gL^−1^	39.4 *μ*gL^−1^	—	—	—
Asante et al. 2012 [59]	Urine/Greater Accra region/	—	—	—	—	1.1 (0.6) *μ*gL^−1^	142 (108) *μ*gL^−1^	2090 (1070) *μ*gL^−1^
Fianko et al. 2007 [61]	Rivers supplying water to Iture Estuary	—	—	—	—	—	—	—
	***Sorowie***	—	—	—	2.1 *μ*gL^−1^	—	—	—
	***Kakum***	—	—	—	1.9 *μ*gL^−1^		—	—
Asante et al. 2007 [15]	Urine (mine workers)/Western region/Tarkwa and its environ	—	404.6 (174–676) *μ*gL^−1^	—	3267.13 (207–8120) *μ*gL^−1^	4.36 (0.8–8) *μ*gL^−1^	—	243.89 (21–545) *μ*gL^−1^
Asante et al. 2007 [15]	Urine (non-mine workers)/Western region/Tarkwa and its environ	—	397.98 (72.7–580) *μ*gL^−1^	—	4394.12 (210–9660) *μ*gL^−1^	8.17 (1–22) *μ*gL^−1^		217.93 (15.6–558) *μ*gL^−1^
Asante et al. 2012 [59]	Urine (e-waste recycling workers)/Greater Accra region/Agbogbloshie	—	0.09 (0.049–0.22) *μ*gL^−1^	—	0.1 (<0.1–0.4) *μ*gL^−1^	<0.1 (<0.1–0.16) *μ*gL^−1^	124 (56.8–197) *μ*gL^−1^	3.85 (1.09–6.75) *μ*gL^−1^

**Table 2:** Levels of metals in water from boreholes by region in Ghana.

Reference	Numbers of boreholes/community	As	Hg	Fe	Mn	Cd	Zn	Cr	Cu	Pb
*Ashanti region *										
Amasa 1975 [31]	Boreholes in selected communities in Obuasi area	2250 *μ*gL^−1^	—	—	—	—	—	—	—	—
Smedley 1996 [62]	4 boreholes in selected communities in Obuasi area	53.75 (14–64) *μ*gL^−1^	—	2046.3 (980–4447) *μ*gL^−1^	220 (79–313) *μ*gL^−1^	—	126.5 (3–226) *μ*gL^−1^	<0.3 *μ*gL^−1^	1.45 (0.7–2.4) *μ*gL^−1^	<0.06 *μ*gL^−1^
Boadu et al. 2000 [25]	Underground water at Konongo old mining shaft	11,950 (11,700–12,200) *μ*gL^−1^	—	—	—	—	—	—	—	—
			—	—	—	—	—	—	—	—
Akabzaa et al. 2007 [24]	Obuasi and its environ		—	—	—	—	—	—	—	—
	8 boreholes in communities within the operating area of AGC	282.7 (15–801) *μ*gL^−1^	2.5 *μ*gL^−1^	2570.7 (21–5342) *μ*gL^−1^	106.14 (43–248) *μ*gL^−1^	<2.0 *μ*gL^−1^	43 (14–117) *μ*gL^−1^	—	46.4 (1–94) *μ*gL^−1^	36.3 (1–96) *μ*gL^−1^
	5 boreholes in communities immediately downstream	<1 *μ*gL^−1^	2.5 *μ*gL^−1^	517.5 (40–1811) *μ*gL^−1^	124.5 (1–329) *μ*gL^−1^	<2.0 *μ*gL^−1^	40.9 (12–127) *μ*gL^−1^	—	19.25 (3–44) *μ*gL^−1^	24 (5–48) *μ*gL^−1^
Akabzaa et al. 2007 [24]	15 bores in the vicinity of Obuasi mine	82 (9–801) *μ*gL^−1^	4 (1–9) *μ*gL^−1^	1343 (19–15340) *μ*gL^−1^	120 (1–329) *μ*gL^−1^	2 (2–9) *μ*gL^−1^	42 (4–127) *μ*gL^−1^	—	25 (1–94) *μ*gL^−1^	16 (<0.01–96) *μ*gL^−1^
Tay and Momade 2006 [26]	67 boreholes, 24 wells in the northern part of the Ashanti Gold belt	—	—	—	—	—	—	—	—	—
		—	205 *μ*gL^−1^	775 *μ*gL^−1^	525 *μ*gL^−1^	10 *μ*gL^−1^	—	—	—	—
	September (light rains)	—	190 (<1.0–520) *μ*gL^−1^	990 (10–2120) *μ*gL^−1^	620 (10–1090) *μ*gL^−1^	10 (1–24) *μ*gL^−1^	—	—	—	—
	June (heavy rains)	—	220 (100–700) *μ*gL^−1^	560 (200–2360) *μ*gL^−1^	430 (10–30) *μ*gL^−1^	10 (<1–76) *μ*gL^−1^	—	—	—	—
Amedjoe et al. 2012 [63]	22 boreholes and hand dug wells in the Obuasi area (wet-June + dry-Feb seasons)	570 (240–1320) *μ*gL^−1^	—	170 (30–480) *μ*gL^−1^	—	—	50 (30–480) *μ*gL^−1^	—	—	—
*Western region *			—	—	—	—	—	—	—	—
Asante et al. 2007 [15] March (Light rains)	12 boreholes in selected communities in Tarkwa area	1.3 (0.5–1.0) *μ*gL^−1^	<0.5 (<0.5–2.3) *μ*gL^−1^	—	797 (2.24–4970) *μ*gL^−1^	0.06 (0.01–0.16) *μ*gL^−1^	10.3 (1.82–24.5) *μ*gL^−1^	0.78 (0.03–2.1) *μ*gL^−1^	4.29 (0.93–11.1) *μ*gL^−1^	0.16 (0.01–0.32) *μ*gL^−1^
Obiri 2007 [64] (August 2004 to June 2005)	4 boreholes in Dumasi in Wassa West District	5142 (4987–6521) *μ*gL^−1^	—	7855 (7520–8515) *μ*gL^−1^	374 (111–586) *μ*gL^−1^	3.35 (2–5) *μ*gL^−1^	6.68 (6-7) *μ*gL^−1^	42 (26–45) *μ*gL^−1^	—	6.56 (5–8) *μ*gL^−1^
Asante et al. 2007 [15] (March 2004)	12 boreholes in selected communities in Tarkwa area	1.3 (0.5–4.0) *μ*gL^−1^	<0.05–2.30 *μ*gL^−1^	—	797 (2.2–4970) *μ*gL^−1^	0.06 (0.01–0.16) *μ*gL^−1^	10.3 (1.82–24.5) *μ*gL^−1^	0.78 (0.46–1.8) *μ*gL^−1^	4.29 (0.93–11.1) *μ*gL^−1^	0.16 (0.01–0.32) *μ*gL^−1^
Armah et al. 2011 [65]	Tarkwa mining area	4 (<0.1–22.0) *μ*gL^−1^	—	144 (<0.1–1499) *μ*gL^−1^	100 (<0.1–1500) *μ*gL^−1^	2 (<0.1–28) *μ*gL^−1^	0.5 (0.4–3.6) *μ*gL^−1^	0.2 (<0.1–1.0) *μ*gL^−1^	0.2 (0.4–717) *μ*gL^−1^	3.7 (0.4–34.1) *μ*gL^−1^
*Eastern Region *										
Kortatsi et al. 2008 [66]	45 boreholes in selected communities in the Eastern region	5.5 (<2–9) *μ*gL^−1^	—	—	—	—	—	—	—	—
Tay and Kortatsi 2008 [47]	Boreholes from 68 communities in the Eastern region		—	—	—	—	—	—	—	—
	22 communities in the Suhum districts			10 (10–400) *μ*gL^−1^	100 (10–500) *μ*gL^−1^		—	—	—	—
	15 communities in the Akim districts			88.6 (10–500) *μ*gL^−1^	135.4 (10–500) *μ*gL^−1^		—	—	—	—
	7 communities in the Akwapim district			40 *μ*gL^−1^	20 *μ*gL^−1^					
*Greater Accra Region *										
Kortatsi et al. 2008 [66]	21 boreholes in selected communities in the Greater Accra region	3.5 (<2–5) *μ*gL^−1^	—	—	—		—	—	—	—
Tay and Kortatsi 2008 [47]	Boreholes in 24 communities in the Ga districts			40 (10–1010) *μ*gL^−1^	40 *μ*gL^−1^		—	—	—	—
*Volta Region *							—	—	—	—
Tay 2007 [67]	34 boreholes at Akatsi area			270 (10–3120) *μ*gL^−1^	330 (10–1450) *μ*gL^−1^		—	—	—	—
	27 boreholes at Ketu area			350 (<1–1510) *μ*gL^−1^	2350 (10–1450) *μ*gL^−1^		—	—	—	—
Kortatsi et al. 2008 [66]	44 boreholes in selected communities in the Volta region	15 (<2.0–28) *μ*gL^−1^								

Reference	Number of boreholes/community	Ni	Co	Cr	Cd	Se	Th	Sb	Sr	Mn

*Ashanti region *										
Smedley 1996 [62]	4 boreholes in selected communities in Obuasi area	1.54 (1–2.4) *μ*gL^−1^	2.36 (0.73–5.57) *μ*gL^−1^	<0.3 *μ*gL^−1^	—	—	—	<0.04 *μ*gL^−1^	277.75 (125–380) *μ*gL^−1^	—
Akabzaa et al. 2007 [24]	15 bores in the Vicinity of Obuasi mine	13 (2–46) *μ*gL^−1^			2 (2–9) *μ*gL^−1^					
Tay and Momade 2006 [26]	67 boreholes, 24 wells in the northern part of the Ashanti Gold belt									
		45 *μ*gL^−1^								
	September (light rains)	<10 *μ*gL^−1^								
	June (heavy rains)	45 (10–80) *μ*gL^−1^								
*Western region *										
Asante et al. 2007 [15] March (**Light rains**)	12 boreholes in selected communities in Tarkwa area		2.5 (0.02–8.9) *μ*gL^−1^	0.78 (0.03–2.1) *μ*gL^−1^					291 (19–1320) *μ*gL^−1^	
Obiri 2007 [64] (August 2004 to June 2005)	4 boreholes in Dumasi in Wassa West District		33.25 (10–50) *μ*gL^−1^		3.5 (2–5) *μ*gL^−1^					
Asante et al. 2007 [15] (March 2004)	12 boreholes in selected communities in Tarkwa area		2.5 (0.07–8.9) *μ*gL^−1^	0.78 (0.03–2.1) *μ*gL^−1^					291.04 (19–1320) *μ*gL^−1^	
Armah et al. 2011 [65]		4 (3) *μ*gL^−1^	—	0.2 (<0.1–1.0) *μ*gL^−1^	2 (<0.1–28) *μ*gL^−1^	—	—	—	—	100 (<0.1–1500) *μ*gL^−1^
Tay 2007 [67]	34 boreholes at Akatsi area									40.79 (1.2–111.7) *μ*gL^−1^
	27 boreholes at Ketu area									47.39 (4.3–183) *μ*gL^−1^
Tay and Kortatsi 2008 [47]	Boreholes in 24 communities in the Ga districts									37400 (7700–306000) *μ*gL^−1^
Tay and Kortatsi 2008 [47]	22 communities in the Suhum districts									11900 (1400–132900) *μ*gL^−1^
Tay and Kortatsi 2008 [47]	15 communities in the Akim districts									12200 (1100–42400) *μ*gL^−1^

**Table 3:** Period of data collection and analytical techniques.

Reference	Period of data collection	Region/community	Pollutant studied	Source of pollutant	Concentration of pollutants	Analytical Technique
Kortatsi et al. 2008 [66]	Not reported	Greater Accra region: 21 communities; Eastern region: 23 communities; Volta region: 44 communities	As	Water samples from 15 selected boreholes	See Tables 1–4	Wagtech Arsenator field test kit

Amonoo-Neizer et al. 1996 [19]	March 1992-1993	Ashanti region: Obuasi and its environ	As, Hg	Soil and food crops^*^, vegetation, and mud fish	See Tables II and III	**As**: UV-visible spectrophotometer A SPEKOL II **Hg**: cold vapour atomic absorption spectrophotometer

Boadu et al. 2000 [25]	Nov 1995, March 1996, July 1996, Nov 1996	Eastern region: Konongo and its surrounding towns and villages; Ashanti region: Odumase, Patriensa and Obenemase	As	Water sample from underground water, river, ponds of a river	See Tables 1 and 2	Instrumental neutron activation analysis

Serfor-Armah et al. 2001 [68]	June 1996–August 1998	Greater Accra region: Prampram, Nungua; Central region: Winneba, Cape Coast; and Western region: Sekondi, Axim	As, Al, Br, Fe, Mn, Cd, Hg, Zn, Ni, V, La	Seaweeds	See Tables III and IV	Neutron activation analysis

Asante et al. 2007 [15]	March 2004	Western region: Tarkwa and its environ	V, Cr, Mn, Co, Cu, Zn, As, Se, Rb, Sr, Mo, Ag, Cd, In, Sn, Sb, Cs, Ba, Hg, TI, Pb, Bi	Water samples from bore boles, wells, rivers/streams and urine samples	Bore hole and well water: <0.1–4.0 *μ*g/L Rivers/stream: 0.5–73 *μ*g/L Urine in mine workers: 34–650 *μ*g/L and in non-workers: 43–700 *μ*g/L	**As**: hydride generation atomic absorption spectrophotometer (HG-ASS); **Hg and Se**: cold vapour atomic Absorption spectrometry; **Other metals**: inductively coupled plasma mass spectrometry

Serfor-Armah et al. 2006 [28]	Jan 2002–April 2003	Western region: Prestea and its environ	As, Sb	Water samples from stream and soil sample	See Tables 2 and 3, Figures 3 and 4	Instrumental neutron activation analysis (INAA)

Rossiter et al. 2010 [69]	July/August 2007	Ashanti region (obuasi and its environs), North of the Volta region, Upper East region (Bolgatanga area)	Al, As, Cd, Co, Cr, Cu, Fe, Mg, Mn, Ni, Pb, Se, U, V, Zn	Water samples from boreholes, wells and rivers	See Table 1	Inductively coupled plasma-optical emission spectroscopy (ICP-OES)

Basu et al. 2011 [57]	Summer 2009	Upper East region (Talensi-Nabdam district: Obuasi, World Bank, Kejitia)	Al, As, Cd, Co, Cr, Cu, Mn, Ni, Pb, Se, Zn	Urine sample	See Table 1	Inductively coupled plasma mass spectrometer (ICPMS)

Adokoh et al. 2011 [70]	Not reported	Western region: Pra Estuary-Shama Central region: Benya Lagoon-Elimina; Fosu Lagoon-Cape Coast; Narkwa Lagoon-near Saltpong Beach	Al, As, Cd, Hg	Water and sediments samples	See Table 3	Neutron activation analysis

Armah et al. 2011 [65]	April 2010	Western region: Tarkwa mining area	As, Mn, Cd, Fe, Pb, Ni	Water samples from boreholes and taps	Table 1	Flame atomic absorption spectrophotometer

Armah et al. 2010 [5]	Feb–June 2009	Ashanti region (Obuasi and its environ)	As, Cu, Mn, Cd, Fe, Pb, Zn	Water samples from taps and surface water bodies	See Table 2	Flame atomic absorption spectrophotometer

Golow et al. 1996 [71]	Not reported	Ashanti region (Obuasi and its environ)	As	Soil samples from villages of varied distances from Obuasi Township	Figures 2 and 3	Schimadzu UV-120-02 spectrophotometer

Obiri et al. 2006 [72]	Not reported	Western region (Bogoso, Prestea, Tarkwa, Tamso)	Hg, Zn, Cd, As	Food crops: cassava, cocoyam, and other tuber crop samples	See Tables III, VII, VIII, XI	Schimadzu atomic absorption spectrophotometer

Essumang et al. 2007 [16]	Not reported	Western region: Tarkwa: Efuantah, Nsuta and Tamso	As, Cd, Hg	Food crop: cocoyam and water cocoyam samples	See Table 1	**As, Cd**: flame atomic absorption spectrophotometer **Hg**: cold vapour technique

Obiri 2007 [64]	August 2004–June 2005	Western region: Dumasi in Wassa West District	As, Zn, Cd, Fe, Co, Cr, Pb, Mn	Water samples from boreholes	See Table 4	Flame atomic absorption spectrophotometer Shimadzu model coupled with an arsine gas generator

Adomako et al. 2011 [73]	Not reported	Selected supermarkets and markets in some regions in Ghana	As, Cd, Co, Cu, Mn, Pb, Se, Zn	Grains	See Figure 2	High performance liquid chromatography (HPLC)

Adomako et al. 2008 [74]	Jan to sept 2005: bimonthly sampling	Ashanti region/River Subin	Al, As, Cd, Cu, Cr, Zn	Water and sediment samples	*Water sample* Tables 3 and 4 *Sediment sample* Tables 3 and 4	Neutron activation analysis

Amasa 1975 [31]	Not reported	Ashanti region (Obuasi and its environ)	As	Urine, Food crops, vegetation, soil, water samples	Tables 1 and 2	Neutron activation analysis

Golow and Adzei 2002 [75]	Not reported	Central region: Dunkwa-on-Offin	Zn	Soil and cassava tuber samples	Figures 2 and 3	AAS/Perkin-Elmer 51000 PV

Golow and Adzei 2002 [75]	Not reported	Central region: Dunkwa-on-Offin	Hg	Soil and cassava tuber samples	Figures 2 and 3	Cold vapour cell in AAS

Oppong et al. 2010 [32]	August 2005, Jan 2006	Central region: Awisam, Twifo Praso, Twifo Mampong Western region: Daboaso, Beposo	Hg	Soil, sediments and, fish samples	Tables 1 and 2	Cold vapour atomic absorption spectrophotometer

Yidana et al. 2008 [76]	NA	Western region: Ankobra basin at Ankwaso, Dominase, Prestea	Hg, Mg	Water samples	Table 1	Not reported

Bentum et al. 2010 [77]	Not reported	Eastern region: Odumase-Atua area	Pb, As, Cd	Breast milk from lactating mothers	**Pb**: 4.83 ± 9.016 *μ*g/L; range: LOD-32.0 ± 1.935 *μ*g/L **As**: 1.54 ± 1.935 *μ*g/L; range: LOD-6.22 *μ*g/L Cd: 1.34 ± 2.194 *μ*g/L range: LOD-12.301 *μ*g/L	Atomic absorption spectrophotometer (Philip AAS 9200u Model)

Essumang 2009 [55]	Not reported	Western region: Tarkwa	As	Human hair samples	0.0142–0.0515 *μ*g/g	Inductively coupled plasma atomic emission spectrometer

Asante et al. 2007 [15]	March 2004	Western Region: Tarkwa and its environ	Cr, Mn, Co, Cu, Zn, Ga, As, Se, Rb, Sr	Water samples from boreholes, rivers/streams and urine sample of mine workers	Tables 1 and 2	Hydride generation-atomic absorption spectrophotometer

Abrefah et al. 2011 [58]	Not reported	Western region: mine workers at Tarkwa	As, Hg	Urine samples	**As** Distance from mine of workers 10 km: 6.76 ± 1.43 *μ*g/L 2 km: 1.78 ± 1.33 *μ*g/L 0.5 km: 8.03 ± 1.75 *μ*g/L Casual: 10.44 ± 1.88 *μ*g/L Gold ore workers: 14.75 ± 1.62 *μ*g/L **Hg** Distance from mine of workers 10 km: 0.36 ± 0.11 *μ*g/L 2 km: 0.47 ± 0.12 *μ*g/L 0.5 km: 0.51 ± 0.16 *μ*g/L Casual: 0.57 ± 0.14 *μ*g/L Gold ore workers: 0.56 ± 0.21 *μ*g/L	Instrumental neutron activation analysis (INAA) for As; ^197^Hg induced radionuclide in INAA for Hg

Kumi-Boateng 2007 [78]	Nov 2007 to Jan 2010 once/month	Ashanti region: Obuasi		Soil sample Sediment sample	Soil sample 0–15 cm: 69.72 ppm; 15–30 cm: 42.90 ppm Sediment sample 34 253 ppm	Atomic absorption spectrophotometer

Fianko et al. 2007 [61]	Weekly from Dec to June	Central region: Iture Estuary	Cd, Pb, Se, Zn	Water sample	See Figures 2–5, Table 6	Atomic absorption spectrophotometer

Basu et al. 2011 [57]	Summer 2009	Upper East region: Obuasi, World Bank, Kejitia	Al, As, Cd, Co, Cr, Cu, Mn, Ni, Pb, Se, Zn	Urine samples	See Table 1	Inductively coupled plasma mass spectrophotometer equipped with a quadrupole analyser and octopole collision

Kwaansa-Ansah et al. 2010 [60]	Not reported	Central region; Dunkwa-on-Offin area	Hg	Urine and hair samples	See Tables 1 and 2	Urine: cold vapour atomic absorption spectrometry using an automatic mercury analyser model HG 5000 Hair: open flask method

Paruchuri et al. 2010 [56]	May and June 2009	Upper East region Bolgatanga	Hg	Urine and hair samples	Urine 170 ± 77.3 *μ*g/g (0.2–708) Hair 0.1 ± 3.2 *μ*g/g (0.0–22.9)	Direct mercury analyzer

Adimado and Baah 2002 [35]	Not reported	Western region: Anwiaso; Sahuma, Tanoso, Elubo	Hg	Blood, urine, hair, nail, and fish samples	See Table 2	Atomic absorption spectrophotometer with flow injection mercury hydride system

Smedley 1996 [62]	Not reported	Ashanti region: Obuasi; Upper East region: Bolangatanga	Cr, Co, Ni, Cu, Zn, Y, Mo, Pb, Rb, Sb, Cs, U, Fe, As, Al	Water samples from boreholes	See Tables 1–10	Not reported

Amonoo-Neizer and Amekor 1993 [14]	Not reported	Ashanti region: Kumasi and Obuasi area	As	Vegetation, cooked food, local fish, and meat (goat) samples	Kumasi: 0.07–7.20 mg/kg Obuasi: 0.12–70.50 mg/kg	

Amedjoe et al. 2012 [63]	June-July; Sept-Oct. 2010	Ashanti region: Homase area	Fe, Zn, As, Mg	Water samples from streams, hand-dug wells, borehole	See Tables 1 and 2	Not reported

Akabzaa et al. 2007 [24]	Not reported	Ashanti region: Obuasi mine	Fe, Mn, Cu, Ni, Zn, Pb, Cd, Hg, As	Water samples from streams, boreholes, hand-dug well	Tables 1 and 2	Not reported

Asante et al. 2012 [59]	Not reported	Greater Accra: Agbogbloshie area in Accra; Ashanti region: Obuasi	V, Cr, Mn, Fe, Co, Cu, Zn, Ga, As, Se, Rb, Sr, Mo, Ag, Cd, In, Sn, Sb, Cs, Ba, Hg, Tl, Pb, Bi	Urine sample	Tables 1 and 2	All: metals: inductively coupled plasma mass spectrometer; Hg: cold vapour atomic absorption spectrometer

Akabzaa et al. 2007 [24]	2002–2004	Ashanti region: Obuasi and its environ	Fe, Mn, Cu, Ni, Zn, Pb, Cd, Hg, As	Water samples from streams, boreholes, hand-dug wells, and fruit samples	See Tables 5.2–5.4	Atomic absorption spectrometry

Tay and Kortatsi 2008 [47]	Feb 2005–Dec 2005	Eastern and Greater Accra regions: 68 communities within Densu basin in these regions	Mg, Fe, Mn	Ground water samples	Tables 2a–2d	Not reported

Karikari and Ansa-Asare 2006 [79]	July 2003 and March 2004	Eastern region: Akwadum, Mangoase, Asuboi, Pakro and Ashalaji	Fe, Mn, Cu, Zn, Pb	Water sample from Densu river	Table 2	Atomic absorption spectrophotometer

Boamponsem et al. 2010 [29]	Sept 2008–Jan 2009	Western region: Teberebie, Mile 7 in the Wassa West District	Sb, Mn, Cu, V, Al, Co, Hg, As, Cd, Th	Water and sediments samples	Tables 2 and 3	Instrumental neutron activation analysis

Tay et al. 2010 [80]	Nov 2003 and Oct 2004	Greater Accra region: Sakumo II and Muni lagoons	Cu, Zn, Pb, Mn, Fe, Cd,	Water and sediments	Tables 1–4	Flame atomization

Kortatsi 2007 [81]	Not reported	Western region: Ankobra basin	Ag, Al, As, B, Ba, Be, Cd, Co, Cr, Cu, Fe, Hg, Li, Mn, Mo, Ni, Pb, Rb, Sb, Se, Sr, Th	Water samples from boreholes in the Ankobra Basin	Tables 1 and 2	ICP-MS

Dapaah-Siakwan and Gyau-Boakye 2000 [82]	June 2002 to March 2003	Ashanti region: Obuasi	As, V, Th, Sb, U, Cr	Lichen samples	Table 2	As, Sb, U: epithermal instrumental neutron analyses Cr, V, Th: thermal instrumental neutron analyses

Nartey et al. 2005 [83]	May-June and Sept to Novem each year	Eastern region: Akwapim area	Zn, Fe, Mn, Cu	Surface water samples from springs/streams	Table 2e	Flame atomic absorption spectrometer

Koranteng-Addo et al. 2011 [84]	Nov 2009 to Jan 2010	Western region: Tarkwa gold mining area	Cu, Zn, Fe, Mn	Sand and clay soil samples	Table 1	ICP-Atomic absorption spectrophotometer

Hayford et al. 2009[30]	Not reported	Western region: Tarkwa and its environs	As, Hg, Sb, V, Cu, Zn, Cr	Samples from cassava, plantain, and soil	Table 1	Instrumental neutron analyses

Ansa-Asare and Asante 2000 [85]	March, May, July, Sept, Nov 1995 and Jan 1996	Eastern region: Birim basin and its environ	Zn, Pb, Cu, Cd, Mn, Fe	Water sample from Birim basin and its environ	Table 4	Atomic absorption spectrophotometry (Varian 1275 AAS)

Bentum et al. 2011 [86]	Nov 2009	Central region: Cape coast	Fe, Cu, Zn, Pb, Al	Sediments sample from Fosu lagoon	Tables 3 and 4	Atomic absorption spectrophotometry (Varian 235 AAS)

Tay et al. 2008 [87]	Nov 2003 and Oct 2004	Greater Accra and Volta regions: James Town, Salaha, Tema Fishing harbour, Sogakope	Fe, Mn, Cu, Pb, Zn, Cd	Fish sample	Table 3	Flame atomization using Unican 969 Atomic absorption spectrophotometer

Nyarko et al. 2006 [34]	June 2002 and March 2003	Ashanti region: Obuasi and its environs	As, V, Th, Sb, U, Cr	Lichen samples	Tables 1 and 2	Neutron activation analysis

Dankwa and Biney 2005 [46]	Aug 1994	Central region: Kaniago, Buabuasin, Kubi, Baadoa and Twifu Praso	Hg, Cd, Pb, As, Cu, Zn, Mn, Fe	Sediment, fish, and water samples		Flame and cold atomic absorption spectrophotometry

Tay 2007 [67]	2002 and 2004	Volta region: Ketu and Akatsi	Mg, Fe, Mn	Water samples from boreholes		Atomic absorption spectrophotometry

Tay and Momade 2006 [26]		Ashanti region: Obuasi area	Ni, Hg, Pb, Mn, Fe, Cd	Water samples from borehole, well, and stream		Atomic absorption spectrophotometry

Donkor et al. 2006 [33]	July 2002	Central, western and Eastern regions: River Pra basins	Hg	Water samples from river pra basin		Ultraclean free-metal sampling protocol

**(a):** 

Reference	Community	As	Hg	Fe	Mn	Cd	Zn	Cr	Cu	Pb
*Ashanti region *										
Boadu et al. 2000 [25]	Rivers at Konongo and surrounding towns and villages: Odumase, Patrienso and Obenemase									
	River Owerri	271 (140–390) *μ*gL^−1^								
	River Awerekye	35 (30–40) *μ*gL^−1^								

Tay and Momade 2006 [26]	10 streams in northern parts of Ashanti Gold belts		32 *μ*gL^−1^	115 *μ*gL^−1^		4.4 *μ*gL^−1^				6 *μ*gL^−1^
	Dry season (Feb 2000)		660 (190–1330) *μ*gL^−1^	1080 (280–2,120) *μ*gL^−1^	185 (70–300) *μ*gL^−1^	9 (<1–13) *μ*gL^−1^		70 *μ*gL^−1^		90 *μ*gL^−1^
	Wet season (June 2000)		320 (100–700) *μ*gL^−1^	1,150 (200–2,360) *μ*gL^−1^	20 (10–30) *μ*gL^−1^	44 (<1–70) *μ*gL^−1^				

Akabzaa et al. 2007 [24]	Obuasi and its environ:	1891 *μ*gL^−1^	1.8 *μ*gL^−1^	1719 *μ*gL^−1^	285.4 *μ*gL^−1^	<0.2 *μ*gL^−1^	12 *μ*gL^−1^		8.6 *μ*gL^−1^	4.6 *μ*gL^−1^
	4 rivers in communities within the operating area of AGC	1113 (<10–3071) *μ*gL^−1^	9.5 *μ*gL^−1^	2935 (358–5603) *μ*gL^−1^	693.7 (472–1525) *μ*gL^−1^	<2 *μ*gL^−1^	30.5 (14–69) *μ*gL^−1^		18.3 (2–27) *μ*gL^−1^	515 *μ*gL^−1^
	3 rivers in communities immediately downstream	6452.5 (310–18910) *μ*gL^−1^	1 *μ*gL^−1^	6912.2 (259–17190) *μ*gL^−1^	913.4 (146–2584) *μ*gL^−1^	2 *μ*gL^−1^	39 (19–120) *μ*gL^−1^		35.3 (12–86) *μ*gL^−1^	105 *μ*gL^−1^

Akabzaa et al. 2007 [24]	Vicinity of Obuasi mine	3137 (9–18910) *μ*gL^−1^	8 (1–18) *μ*gL^−1^	5032 (259–17190) *μ*gL^−1^	758 (146–2584) *μ*gL^−1^	2 *μ*gL^−1^	34 (3–120) *μ*gL^−1^		23 (<0.1–86) *μ*gL^−1^	14 (1–57) *μ*gL^−1^

Adomako et al. 2008 [74]	River Subin	13.9 (07–160) *μ*gL^−1^				12.5 (2–50) *μ*gL^−1^	6600 (4280–10200) *μ*g^−1^	6 (10–19) *μ*gL^−1^	1330 (1320–7040) *μ*gL^−1^	

Armah et al. 2010 [5]		434 *μ*gL^−1^; IQ-UQ (136–1135) *μ*gL^−1^	302 *μ*gL^−1^; IQ-UQ (2–646) *μ*gL^−1^	705 *μ*gL^−1^; IQ-UQ (242–1208) *μ*gL^−1^	892 *μ*gL^−1^; IQ-UQ (459–2152) *μ*gL^−1^	674 *μ*gL^−1^; IQ-UQ (418–1350) *μ*gL^−1^	139 *μ*gL^−1^; IQ-UQ (3–667) *μ*gL^−1^		603 *μ*gL^−1^; IQ-UQ (245–1831) *μ*gL^−1^	0.69 UQ (0.06–2.10) *μ*gL^−1^

Amedjoe et al. 2012 [63]		41 (39) *μ*gL^−1^		127 (98) *μ*gL^−1^			2 (1) *μ*gL^−1^		2.5 (2.3) *μ*gL^−1^	

*Western region *										
Serfor-Armah et al. 2006 [28]		384 (33) *μ*gL^−1^								

Kortatsi 2006 [88]		300 (1000) *μ*gL^−1^	4600 (3700) *μ*gL^−1^	170900 (306500) *μ*gL^−1^	41900 (36400) *μ*gL^−1^		168000 (38700) *μ*gL^−1^	300 (100) *μ*gL^−1^	2400 (4100) *μ*gL^−1^	1100 (1400) *μ*gL^−1^

Asante et al. 2007 [15]		18 (0.5–73) *μ*gL^−1^			682 (11.1–2530) *μ*gL^−1^	0.04 (0.04–0.08) *μ*gL^−1^	138 (3.43–1500) *μ*gL^−1^	0.52 (0.10–1.2) *μ*gL^−1^	1.3 (0.97–9.17) *μ*gL^−1^	0.85 (0.02–3.71) *μ*gL^−1^

Asante et al. 2007 [15]		18 *μ*gL^−1^	BDL		682 *μ*gL^−1^	0.04 *μ*gL^−1^	138 *μ*gL^−1^	0.52 *μ*gL^−1^	2.65 *μ*gL^−1^	0.85 *μ*gL^−1^

Boamponsem et al. 2010 [29]	**Tarkwa gold mining district**									
	Angonabeng	1220 *μ*gL^−1^	148 *μ*gL^−1^		1780 *μ*gL^−1^	1110 *μ*gL^−1^				
	Bediabewu	52.2 *μ*gL^−1^	52.2 *μ*gL^−1^		804 *μ*gL^−1^	1110 *μ*gL^−1^				

Adokoh et al. 2011 [70]		0.016 *μ*gL^−1^	0.002 *μ*gL^−1^			0.016 *μ*gL^−1^				

Armah et al. 2011 [65]	Abunpuni Angonabeng Adisakrom Bediabewu Domeabra Nkwantakrom Teberebie	4 (4.1) *μ*gL^−1^		144 (279.2) *μ*gL^−1^	100 (228.3) *μ*gL^−1^	2 (2.9) *μ*gL^−1^				5.7 (0.3) *μ*gL^−1^

**(b):** 

Reference	Community	Al	Ni	Co	V	Cr	Se	Sb	Sr	Mg	Rb
*Western region *											
Serfor-Armah et al. 2006 [28]								14 (0.1) *μ*gL^−1^			

Kortatsi 2006 [88]		12700 (47200) *μ*gL^−1^	1100 (1400) *μ*gL^−1^		100 (100) *μ*gL^−1^	300 (100) *μ*gL^−1^	300 (100) *μ*gL^−1^		27900 (22000) *μ*gL^−1^	7900 (4300) *μ*gL^−1^	

Asante et al. 2007 [15]				1.3 (1.9) *μ*gL^−1^	0.46 (0.57) *μ*gL^−1^	0.52 (0.34) *μ*gL^−1^			214 (359) *μ*gL^−1^		4.65 (3.68) *μ*gL^−1^

Asante et al. 2007 [15]				1.3 (0.05–6.9) *μ*gL^−1^	0.46 (0.02–2.0) *μ*gL^−1^	0.52 (0.10–1.2) *μ*gL^−1^	BDL	2.0 (<0.01–15) *μ*gL^−1^	214 (22.6–2240) *μ*gL^−1^		4.65 (1.93–15.5) *μ*gL^−1^

Boamponsem et al. 2010 [29]	**Tarkwa gold mining district**										
	Angonabeng			409 *μ*gL^−1^	37 *μ*gL^−1^			4140 *μ*gL^−1^			
	Bediabewu			230 *μ*gL^−1^	37.8 *μ*gL^−1^			1070 *μ*gL^−1^			

Adokoh et al. 2011 [70]		0.964 *μ*gL^−1^									

Armah et al. 2011 [65]	Abunpuni Angonabeng Adisakrom Bediabewu Domeabra Nkwantakrom Teberebie		0.4 (0.3) *μ*gL^−1^								

**(c):** 

Reference	Community	*μ*gL^−1^								
		As	Hg	Fe	Mn	Cd	Zn	Cr	Cu	Pb
*Eastern region *										
Karikari and Ansa-Ansare 2006 [79]	Densu Basin covering Akwadum, Mangoase, Asuboi, Pakro and Ashalaja			964 (614–1190)	291 (264–337)		57 (14–100)		80 (28–274)	18 (<5–39)

Ansa-Asare and Asante 2000 [85]				61.4 (5–157)	60.7 (2–263)	1.4 (<1–2.6)	68.6 (1–89.2)			1.4 (0.1–2.6)

Boadu et al. 2000 [25]	Konongo and its surrounding areas: Odumase, Patriensa, Obenemase									
	River Owerri	27.3 (13–43)								
	River Awirekye	3.5								

Nartey et al. 2005 [83]	Seven streams in the Akwapim Ridge (Otobri, Kobi, Opiafo, Amankrate, Kwati, Ademi, Elemi)			714.3	614.3		200			

*Central region *										
Dankwa and Biney 2005 [46]				2.09		<0.01				0.47

Donkor et al. 2006 [33]			20536							

Fianko et al. 2007 [61]	Rivers supplying water to Iture Estuary									
	***Sorowie***					4	180			6.45
	***Kakum***					3.2	200.1			2.65

Boamponsem et al. 2010 [29]	Kakum	1			145					

**Adokoh et al. ** **2011** [70]	***Benya Lagoon***	0.106	0.003		1.706	0.051				
	***Fosu lagoon***		0.002		1.477	0.036				
	***Narkwa Lagoon***	0.068	0.002		1.112	0.0425				

**Bentum et al.** **2011** [86]	***Fosu Lagoon***			1150	2230		20.9		26.4	

**Tay et al. ** **2010** [80]	Greater Accra			1.65 (0.20)	0.942 (0.06)	0.006 (0.001)	0.113 (0.010)		0.268 (0.06)	0.02 (0.0)

**(a):** 

Reference	Region/community	Source of pollutants studied	As (mgkg^−1^)	Hg (mgkg^−1^)		As (mgkg^−1^)	Hg (mgkg^−1^)		As (mgkg^−1^)	Hg (mgkg^−1^)
	
	*Ashanti region *									
Amasa 1975 [31]	Obuasi and its environ									
		Soil	19.30 (11.75–2875.0)							
		cassava	1.09 (0.83–2.65)							
		Cocoyam	2.16 (1.89–4.80)							
		Plantain	0.62 (0.60–0.63)							
		Orange	2.29							
		Sugar cane	14.75 (14.54–14.96)							
		Palm tree	2875 (2850–2900)							
		Fern	2729 (1100–4700)							
		Bananas	13.46 (11.6–20.9)							

Amonoo-Neizer et al. 1996 [19]	Obuasi and its environ:									
	Villages up to about 2 km from Pompora Treatment Plant (PTP)				Villages up to about 5 km from Pompora Treatment Plant (PTP)			Villages up to about 9 km from Pompora Treatment Plant (PTP)		
		Soil	24.76 (23.6–48.9)	0.56 (0.4–0.7)		3.09 (2.7–3.7)	0.66 (0.66–1.2)		2.61 (2.4–3.3)	0.6
		Plantain	3.40 (3.1–4.3)	0.78 (0.70–1.5)		1.49 (1.4–1.7)	0.6 (0.38–0.9)		1.02 (1.0–1.4)	0.11 (0.1–0.4)
		Cassava	2.6 (1.9–3.3)	1.1 (0.9–1.3)		1.21 (1.0–1.3)	0.40 (0.3–0.5)		1.04 (0.8–1.5)	0.2
		Mud fish				0.42 (0.3–0.9)	0.32 (0.2–0.8)		0.56 (0.5–0.7)	0.24 (0.1–0.4)
		Fern	23.9 (22.8–78.7)	2.57 (2.4–2.6)		3.79 (3.2–30.4)	3.33 (1.8–4.4)		3.83 (2.8–27.8)	1.39 (1.2–4.1)
		Elephant grass	15.2 (14.3–15.6)	3.20 (3.0–3.4)		1.5 (0.9–2.1)	1.1 (0.7–1.5)			
	Villages up to about 4 km from Pompora Treatment Plant (PTP)				Villages up to about 7 km from Pompora Treatment Plant (PTP)			Villages > 9 km from Pompora Treatment Plant (PTP)		
		Soil	3.66 (2.9–30.7)	0.74 (0.3–1.4)		2.79 (2.4–3.6)	1.34 (1.3–2.5)		2.52 (2.1–2.79)	0.56 (0.4–1.2)
		Plantain	1.53 (1.4–3.6)	0.13 (0.1–1.4)		1.35 (1.1–1.6)	0.25 (0.2–0.7)		0.9 (0.5–1.32)	0.41 (0.3–2.1)
		Cassava	1.26 (1.1–2.5)	0.22 (0.2–1.8)		0.91 (0.8–1.1)	0.59 (0.5–0.8)		0.89 (0.7–1.0)	0.34 (0.2–2.5)
		Mud fish	0.45 (0.3–2.7)	0.47 (0.2–2.0)		0.48 (0.4–1.2)	0.22 (0.2–0.6)		0.57 (0.5–0.6)	0.42 (0.4–0.9)
		Fern	5.35 (3.2–50.2)	3.61 (2.1–9.7)		3.53 (2.7–4.5)	1.7 (1.3–2.1)		2.33 (2.1–24.5)	2.6 (2.0–8.0)
		Elephant grass	3.02 (2.0–27.4)	0.38 (0.2–1.8)		1.43 (1.41–1.57)	1.28 (1.0–1.9)		6.5 (6.0–8.0)	5.3 (4.7–5.9)

**(b):** 

Reference	Region/community	Source of pollutants studied	(mgkg^−1^)									
			As	Hg	Fe	Mn	Cd	Zn	Cr	Cu	Ni	Pb
	*Ashanti region *											
Akabzaa et al. 2007 [24]	Obuasi and its environ:	Sediments										
	Communities within the operating area of AGC		1332.26 (0.25–7591.58)	0.83 (0.28–3.02)	34406 (6220–50,180)	47.15 (2.76–167.24)		29.73 (8.87–80.35) mgkg^−1^		25.25 (3.65–80.46)	10.33 (3.28–23.11)	0.65
	Communities immediately downstream		2984.27 (190.38–5778.15)	2.02 (1.44–2.6)	19810 (13910–25710)	15.22 (12.72–24.55)	0.24	102.13 (61.74–142.55)		45.86 (41.44–50.27)	10.22 (8.36–12.08)	60.04 (4.71–115.37)
		Fruits	4.94	0.023	1.71	0.379	0.083	16.55		0.55		0.072
	Communities within the operating area of AGC		4.94 (0.02–12.26)	0.023 (0.02–0.026)	1.71 (0.21–4.11)	0.38 (0.27–0.56)	0.09 (0.04–0.13)	16.55 (7.4–24.30)		0.55 (0.40–0.84)		0.072

Adomako et al. 2008 [74]	Sediment in Subin River in Kumasi/Ashanti region	Sediments	3.43 (2.34–7.65)				1.06 (0.28–1.42)	18.50 (14.40–98.30)	30.57 (18.50–136.40)	3.32 (3.11–12.63)		

Golow et al. 1996 [71]	Obuasi mining area	Top soil	33.3 (25–45)									

Golow et al. 1996 [71]	Obuasi mining area	Fish	0.45 ± 0.12	0.47 ± 0.05								

Nyarko et al. 2006 [34]	Obuasi gold mining area	Lichens	60.95 (6.80–196.0)						6.60 (1.80–14.0)			

**(c):** 

Reference	Region/community	Source of pollutants studied	Sb (mgkg^−1^)	V (mgkg^−1^)	Th (mgkg^−1^)
	*Ashanti region *				
Amonoo-Neizer and Amekor 1993 [14]	Obuasi area	**Uncooked food**			
		cassava	2.55 (1.85–3.25)		
		Cocoyam	2.26 (1.36–3.18)		
		Plantain	3.43 (2.36–4.5)		
		Pepper	2.96 (2.05–3.87)		
		Orange	3.46 (3.10–3.91)		
		Beans	0.99 (1.21–0.77)		
		Pear	1.59 (0.97–2.21)		
		**Cooked food**			
		Cassava	2.67		
		Plantain	3.39		
		Fufu	2.37		
		Oil Palm fruit	3.03		
		Star grass	6.67		
		Elephant grass	4.85		

Amonoo-Neizer and Amekor 1993 [14]	Kumasi area	**Uncooked food**			
		cassava	1.03 (0.84–1.87)		
		Cocoyam	0.97 (0.81–1.13)		
		Plantain	1.10 (0.85–1.35)		
		Pepper	0.58 (0.5–0.66)		
		Orange	0.85 (0.45–1.25)		
		Beans	0.52 (0.42–0.62)		
		Pear	0.76 (0.9–0.62)		
		**Cooked food**			
		Cassava	1.91		
		Plantain	3.03		
		Fufu	1.4		
		Oil Palm fruit	3.5		
		Tobacco	2.14		
		Cocoa	2.44		
		Star grass	6.67		
		Elephant grass	4.85		

Nyarko et al. 2006 [34]	Obuasi gold mining area	**Lichen**		27.63 (2.90–74.80)	1.08 (0.13–4.44)

Serfor-Armah et al. 2006 [28]	Prestea and its environ	Sediment	12.46 (8.50–90.40)		

Hayford et al. 2009 [30]	Tarkwa and its environs	Soil	2.98	188.40	
		Cassava	0.03	0.8	
		Plantain	0.02	0.42	

Boamponsem et al. 2010 [29]	Angonabeng	Sediment	38.9	133	1.64
	Bediabewu	Sediment	309	417	15.72

**(d):** 

Reference	Region/ community	Source of pollutants studied	As	Hg	Fe	Mn	Cd	Zn	Cr	Cu	Ni	Pb
	*Western region *											
Donkor et al. 2006 [33]	Lower Pra river basin	River sediments		25.89 (6.52–57.32) mgkg^−1^								
		Soil		75.61 (3.40–202.32) mgkg^−1^								

Serfor-Armah et al. 2006 [28]	Prestea and its environ	Sediments	2261.8 (942–10,200) mgkg^−1^									
Essumang et al. 2007 [16]	Food crops from Efuantah, Nsuta and Tamso in Tarkwa district	Cocoyam Water cocoyam	146 mgkg^−1^ 383.5 mgkg^−1^	3 mgkg^−1^ 3.5 mgkg^−1^			43 mgkg^−1^ 181.5 mgkg^−1^					

Hayford et al. 2009 [30]	Tarkwa and its environs	Soil	0.7 mgkg^−1^	0.25 mgkg^−1^				38.42 mgkg^−1^	0.72 mgkg^−1^	66.39 mgkg^−1^		
		Cassava	0.43 mgkg^−1^	0.41 mgkg^−1^				26.62 mgkg^−1^	0.44 mgkg^−1^	33.61 mgkg^−1^		
		Plantain	0.34 mgkg^−1^	0.36 mgkg^−1^				18.87 mgkg^−1^	2.2 mgkg^−1^	5.44 mgkg^−1^		

Boamponsem et al. 2010 [29]	Sediments in 5 streams in Tarkwa gold mining district	Sediments										
	Angonabeng		5.41 mgkg^−1^	0.212 mgkg^−1^		36468 mgkg^−1^	0.658 mgkg^−1^			13 mgkg^−1^		
	Bediabewa	“	13.4 mgkg^−1^	0.054 mgkg^−1^		365 mgkg^−1^	0.312 mgkg^−1^			20.1 mgkg^−1^		
	Mile 7 spring		1.65 (1.15–2.22) mgkg^−1^			55.9 (42.1–69.7) mgkg^−1^				2.92 mgkg^−1^		
	Teberebie spring 2		1.94 (0.94–2.94) mgkg^−1^	0.06 mgkg^−1^		189.2 (134.3–244.1) mgkg^−1^	0.20 mgkg^−1^			230.2 mgkg^−1^		
	Teberebie spring 3		1.06 (0.79–1.33) mgkg^−1^	0.01 mgkg^−1^		117770 mgkg^−1^	0.43 mgkg^−1^			1201 mgkg^−1^		

Oppong et al. 2010 [32]	Soil, sediments and fish from the River Pra Basin at Daboaso, Beposo, Twifo Praso and Twifo Mampong											
		**Daboase**										
		Sediment		0.707 mgkg^−1^								
		Soil		0.075 mgkg^−1^								
		Fish		0.166 mgkg^−1^								
		**Beposo**										
		Sediment		0.575 mgkg^−1^								
		Soil		0.145 mgkg^−1^								
		Fish		0.153 mgkg^−1^								

Adokoh et al. 2011 [70]	Sediments in Pra Estuary at Shama	Sediment	0.016 mgkg^−1^	0.017 mgkg^−1^								

Koranteng Addo et al. 2011 [84]	Abandoned open pit in the Tarkwa mining district	Sand			53151.28 (145–119,166) mgkg^−1^	34.45 (26.67–1595) mgkg^−1^		21.06 (4.17–43.17) mgkg^−1^		12 (6.5–56.17) mgkg^−1^		
		Clay			71013.08 (67,000–134,833.3) mgkg^−1^	56.76 (36.16–147.3) mgkg^−1^		20.35 (15–27.67) mgkg^−1^		13.56 (9.17–21.83) mgkg^−1^		

**(e):** 

Reference	Region/ community	Source of pollutants studied	As	Hg	Fe	Mn	Cd	Zn	Cu	Al	Pb
	*Central region *										
Golow et al. 1996 [71]	Dunkwa-on-Offin										
		Top soil (0–5 cm deep)		153.75 mgkg^−1^							
		Cassava leaves		15.63 mgkg^−1^							
		Cassava flesh		6.5 mgkg^−1^							
Golow and Adzei 2002 [75]	Dunkwa-on-Offin										
		Top soil (0–5 cm deep)						41.75 mgkg^−1^			
		Cassava leaves						13.75 mgkg^−1^			
		Cassava flesh						100 mgkg^−1^			
Dankwa and Biney 2005 [46]	Five sites in Offn river basin: Kaniago, Buabuasin, Kubi, Baadoa, Twifo-Praso	Bottom sediment	<500 mgkg^−1^	<200 mgkg^−1^	15.75 × 10^6^ mgkg^−1^	1800 mgkg^−1^	<200 mgkg^−1^	12900 mgkg^−1^	<200 mgkg^−1^		
		**Fish fauna**									
		*Brycinus nurse* R	<300 mgkg^−1^	320 mgkg^−1^	3760 mgkg^−1^	500 mgkg^−1^	<100 mgkg^−1^	2560 mgkg^−1^	300 mgkg^−1^		250 mgkg^−1^
		*Chrysichthys nigrodigitatus* L	<300 mgkg^−1^	230 mgkg^−1^	3310 mgkg^−1^	570 mgkg^−1^	<100 mgkg^−1^	570 mgkg^−1^	2500 mgkg^−1^		440 mgkg^−1^
		*Tilapia zillii* G	<300 mgkg^−1^	60 mgkg^−1^	2660 mgkg^−1^	1350 mgkg^−1^	<100 mgkg^−1^	1820 mgkg^−1^			70 mgkg^−1^
Donkor et al. 2006 [33]	Offin river basin	River sediments		23 (2.73–49.86) mgkg^−1^							
		Soil		263.79 (1.56–2146.96) mgkg^−1^							
Boamponsem et al. 2010 [29]	Kakum	Sediments	1.31 mgkg^−1^			777 mgkg^−1^			3.21 mgkg^−1^	15836 mgkg^−1^	
Adokoh et al. 2011 [70]	Benya Lagoon-Elimina; Fosu Lagoon-Cape Coast; Narkwa Lagoon-near Saltpong Beach	**Fosu Lagoon**									
		Sediments	0.529 mgkg^−1^	0.014 mgkg^−1^			0.526 mgkg^−1^			56.453 mgkg^−1^	
		**Narkwa Lagoon**									
		Sediments	0.603 mgkg^−1^	0.008 mgkg^−1^			0.041 mgkg^−1^			17.925 mgkg^−1^	
		**Benya Lagoon**									
		sediment								60.407 *μ*gg^−1^	
Bentum et al. 2011 [86]	Fosu Lagoon	Sediments			1150 mgkg^−1^			20.9 mgkg^−1^	26.4 mgkg^−1^	2230 mgkg^−1^	28.1 mgkg^−1^

**(f):** 

Reference	Region/community	Source of pollutants studied	As	Hg	Fe	Mn	Cd	Zn	Cr	Cu	Ni	Pb
	*Greater Accra region *											
Tay and Kortatsi 2008 [47]		Shell fish			20.92 (9.68–35.04) mgkg^−1^	22.08 (7.27–22.16) mgkg^−1^	0.26 (0.11–0.34) mgkg^−1^	13.52 (6.55–16.09) mgkg^−1^		1.36 (0.87–7.73) mgkg^−1^		0.084 (0.08–0.44) mgkg^−1^
		Fin fish			14.95 (0.75–23.18) mgkg^−1^	0.036 (0.03–19.37) mgkg^−1^	0.081 (0.08–0.14) mgkg^−1^	11.13 (6.2–19.19) mgkg^−1^		0.244 (0.1–14.18) mgkg^−1^		0.754 (0.9–1.09) mgkg^−1^
Tay et al. 2010 [80]	Sakumo II and Muni lagoons	Sediment			3208.6 (563.2–7486.9) mgkg^−1^	298.21 (63.7–668.2) mgkg^−1^	0.70 (<0.25–0.88) mgkg^−1^	155.2 (10.4–155.2) mgkg^−1^				37.4 (3.79–37.4) mgkg^−1^
